# In Memoriam

**Published:** 1886-05-20

**Authors:** 


### IN MEMORIAM.

At a called meeting of the Atlanta Society of Medicine, held April 26th, the following resolutions were adopted:

Whereas, our esteemed professional brother, Dr. N. O. Harris, has recently met with the loss, by death, of a loving and devoted wife; therefore be it

Resolved, That we, the members of the Atlanta Society of Medicine, of which he was an active and honored officer, do hereby tender to him, in this hour of his affliction, our sincere sympathy and condolence, and the assurance that our hearts beat as one in grief for the irreparable loss which he has sustained.

Resolved, That these resolutions be presented to Doctor Harris and published in the medical journals and daily papers of this city.

W. S. ELKIN,<br>

ARCH. AVARY, } Committee.<br>

V. O. HARDON,

